# Health risk associated with airborne asbestos

**DOI:** 10.1007/s10661-015-4614-3

**Published:** 2015-06-13

**Authors:** Adam Pawełczyk, František Božek

**Affiliations:** Wroclaw University of Technology, ul. Wyspianskiego 27, 50-370 Wroclaw, Poland; University of Defence, Kounicova 65, 662 10 Brno, Czech Republic

**Keywords:** Air pollution, Asbestos, Chronic exposure, Health risk

## Abstract

The following paper presents an assessment of health risks associated with air polluted with respirable asbestos fibers in towns of southwest Poland. The aim of the work was to determine whether or not any prevention measures are necessary in order to reduce the level of exposure to the pollutant. The risk assessment was carried out based on the air analyses and the latest asbestos toxicity data published by the Environmental Protection Agency (US EPA), USA and Office of Environmental Health Hazard Assessment (OEHHA). It was found that in some sites, the concentration of the asbestos fibers exceeded the acceptable levels, which should be a reason of special concern. The highest concentration of asbestos was found in town centers during the rush hours. In three spots, the calculated maximum health risk exceeded 1E-04 which is considered too high according to the adopted standards. So far, it has not yet been possible to find a reasonable method of ensuring the hazard reduction.

## Introduction

Asbestos is a common name that applies to minerals derived from the group of serpentines and amphiboles composing hydrated calcium, magnesium, and sodium silicates. Asbestos has unique chemical and physical properties, such as the resistance to high temperature, resistance to the influence of bases, acids, sea water, etc. In the early years of the past century, asbestos found application in over one thousand production technologies and in about three thousand products. It was used in the manufacture of textile products, yarn, rope, sealing, and abrasive products such as brake blocks, in hydroinsulation, the floor tiles, as the filtration aid in brewing, etc. (Trefler et al. 2004). Nevertheless, it was mostly used in the building industry in the form of asbestos–cement roofing, the so-called eternit, asbestos–cement boards, pipes, and other products. For instance, during the period of several years of the product application in Poland, about 14.5 million tons of asbestos and asbestos containing materials was accumulated (Programme 2010).

First health concerns related to asbestos exposure emerged in the early 1900s. Along with the ever-increasing number of applications, growing evidence revealed carcinogenic properties of asbestos thus motivating authorities to undertake measures in order to protect workers against asbestos-related diseases. In 1930s in England, ventilation and exhaust systems were installed to reduce the exposure. In subsequent years, when the problem became more recognized, exposure standards and legislations were established leading to a ban on asbestos use (Wagner et al. 1960; ACS 2015).

The biggest asbestos health hazard results from the erosion of the already installed, improperly taken down or stored asbestos as well as from the products that contain it. The erosion of these materials has led to the contamination of air with airborne particles, most being respirable fibers.

In 1991, EU member states were obliged to limit the application of asbestos. Today, all European Union member states have a ban on the use and production of asbestos containing materials. At the same time, particular countries have developed programs aiming at the abatement of environment contamination.

A lot of asbestos neutralization methods can be found in literature. The proposed ideas comprise collection of wastes in specially constructed storage yards, cementation, covering the asbestos constructions on site with protective preparations preventing the fibers from release to the environment, destruction using thermal or microwave treatment, etc. (ATON 2014; Block 1998; Debailleul 2002; Mirick and Forrister 1993; Pritchett 1997; Trefler et al. 2003; Zaremba et al. 2010; Zaremba et al. 2011).

According to WHO (2014) estimates, more than 107,000 people die each year from asbestos-related lung cancer, mesothelioma, and asbestosis resulting from occupational exposure. Until recently, there have not been any available precise toxicological factors that could be used to determine the health risk resulting from the exposure to asbestos. Air purity standards have been established; they however do not provide quantitative data on the health hazard. Such information provides the health risk calculations mainly based on the inhalation unit risk and the carcinogenic slope factors published by the OEHHA and US EPA. As the air analyses are mainly expressed in fiber counts per cubic centimeter or meter (*f*/m^3^), the risk is calculated based on the unit risk data related to the number of fibers. On the other hand, the asbestos carcinogenic slope factor is expressed in (mg·kg^-1^·day^-1^)^-1^. Thus, to make the risk assessment calculations possible, asbestos concentration in the air should be converted from f/m^3^ into mg/m^3^. This involves some complications in calculations and additional uncertainty factors in risk assessment evaluation. Usually, the correlation between phase contrast microscopy (PCM) fiber counts and transmission electron microscopy (TEM) mass measurements is very poor. This is due to the fact that certain fiber dimensions have to be assumed to make the conversion possible. In fact, airborne fibers found in the environment are not uniform and differ substantially in length and diameter.

As we made the measurements using the phase contrast microscopy, unit risk value based on fiber counts made by the PCM was used to calculate the risk. This calculated risk is related to the additive combined risk of lung cancer and mesothelioma. It should be noted that TEM can be more appropriate for asbestos measurements while PCM is a nonspecific technique and measures any fibrous material in the air. Thus, the measurements must be viewed and interpreted cautiously (Breslow et al. 1984).

## Materials and methods

### Analyses

For the purpose of the health effects assessment, methodological principles of the health risk assessment related to polluted areas have been applied, based on US EPA guidelines for “Superfund” project (EPA 1980). Foundations of the methodology have been published in the Risk Assessment Guidance for Superfund (EPA 1989). Also, guidelines developed by the Agency for Toxic Substances and Disease Registry were taken into account (ATSDR 2005). The health risk assessment was based on the measurements of asbestos fibers concentration and asbestos toxicity data.

Special attention was paid to the air sampling prior to analyses of asbestos fibers. Since the concentration of fibers in the air strongly depends on the wind, air humidity, temperature, etc., similar weather conditions were chosen for collecting the samples whenever it was possible. The work was done during fine, dry, and windless weather, thus ensuring uniform sampling conditions for all sites. In general, average fiber concentration during the exposure time should be used for the health risk calculations. The problem is that there are not any known precise methods of determination of the average concentrations. For that reason, special approach was applied in this work, namely not average but maximum and minimum concentrations were determined at each spot. In this way, maximum and minimum possible health risks expected at each sampling spot could be derived from the measurements. Calculations based on maximum concentrations mean that a conservative approach in the risk assessment was applied. In that way rather overestimated risks are produced which is a safer approach in the risk assessment.

It is obvious that the maximum concentrations of asbestos fibers are found in the air during rush hours and the minimum ones occur at nights. For that reason, the sampling at each measurement spot was conducted in the early morning between 6 and 8 a.m. and in the afternoons between 4 and 6 p.m. Additionally, one more sampling was carried out at midnights between 11 p.m. and 1 a.m. The sampling spots were chosen taking into account possible highest traffic density in the towns, which means they were located in the neighborhood of busy crossroads in the town centers.

The air samples were collected according to adapted Polish Standard (PS 1984) using Quick Take 30 aspirator with an electronic flow control adjustment at the flow rate of 16.0 dm^3^/min and with 110 min sampling time applied. The air stream was filtered on Sartorius cellulose nitrate membrane filters type 113, with 25 mm diameter and 0.8-μm pores. The filters were protected in boxes and delivered to the laboratory after sampling.

Prior to the microscopic examinations, the filters were treated with diethyl oxalate/dimethyl phthalate balsam in order to make them transparent. The fibrous pollutants were identified and counted using the phase-contrast optical microscopy PCOM, according to (PS 1988). The fiber counts from the measurements composed a basis for further health risk assessment.

### Hazard identification

The asbestos structures produce suspended dust particles and fibers that are inhaled by people and animals. Exposure to the fibers can develop health effects which intensity depends on the following:

- The concentration of asbestos fibers in the air,
- Duration of the exposure period,
- Frequency of the exposure,
- The size of the asbestos fibers inhaled,
- The time since the exposure started, and
- Type of asbestos fibers in the air.

Breathing high levels of asbestos may result in a slow buildup of scar-like tissue in the lungs and in the membrane that surrounds the lungs. The respirable fibers that cause the most problems are the microscopic variety that are small enough to be inhaled deeply into the lungs, having a length exceeding 5 μm and diameter less than 3 μm. Some studies report that the fibers less than 1 μm in diameter and more than 8 μm are the most dangerous (Krakowiak et al. 2009).

There are two types of cancer caused by the exposure to asbestos: cancer of the lung tissue itself and mesothelioma, a cancer of the membrane that surrounds the lung and other internal organs (NCI 2011). The lung cancer and mesothelioma do not develop straight away after the initial exposure. They appear after many years. Smoking, combined with inhaled asbestos does greatly increase the risk of developing lung cancer, but the epidemiological data show that they do not interact with regard to mesothelioma (ATSDR 2011; RAIS 2011; Salvatore et al. 2006; O'Reilly et al. 2007; EPA 2015b).

It has been proved that different types of asbestos fibers vary in relative carcinogenic relative. It appears, for example, that the risk of mesothelioma is greater with exposure to crocidolite than with amosite or chrysotile exposure alone. Also, differences in fiber size distribution may contribute at least as much to the observed variation in risk as does the fiber type itself (ATSDR 2001; IRIS 2011).

In Poland, the asbestos occupational standards in the workplace limit the permissible fiber concentration to 1 mg/m^3^ of the total dust containing chrysotile asbestos and 0.2 asbestos fibers in 1 cm^3^. For crocidolite asbestos, the respective values are 0.5 mg/m^3^ and 0.2 fibers in 1 cm^3^ (MLSP 2002).

The official standards for the municipal air have not been set in Poland, but it was generally agreed on the basis of literature data that 1000 fibers/m^3^ (0.001 fibers/cm^3^) can be accepted as an upper concentration safety level. On the other hand, permissible concentrations of asbestos in the air for certain periods of exposure were established (ME 1998). For 30 min, it amounts to 2.350 mg/m^3^, for 24 h to 1000 mg/m^3^ while for 1 year exposure to 250 mg/m^3^. The first and the third values can only be used for computational purposes.

In the US, asbestos standards were changed in 1994. The new standard was changed significantly. The permissible exposure limit (PEL) had been set to 0.1 fibers/cm^3^ (8 h time weighted average) for any form of asbestos. Thus, the US standard became one of the strictest in the world (LHC 1995; Brownson et al. 2012).

On the other hand, there is no consistent, convincing evidence that ingested asbestos is hazardous. Therefore, the established maximum acceptable concentrations of asbestos in drinking water are not that much restrictive. Maximum contaminant level (MCL) in drinking water for asbestos fibers longer than 10 μm is 7 MFL (million fibers per liter) (EPA 2011a).

### Determination of dose-response relation

The non-carcinogenic effects related to exposure to asbestos are not as evident as carcinogenic ones. Therefore, the health effect considerations were limited just to the cancer risk resulting from the contact with the asbestos polluted environment. According to carcinogen classification, asbestos, CAS number 1332-21-4, is included into group A—human carcinogen (EPA 1986) or into group 1—carcinogenic to humans (IARC 2011). Tables 1 and 2 give toxicity values for asbestos.

**Table 1:** Toxicity data of asbestos (EPA 2011b; OEHHA 2011 and RAIS 2011)

Authority	Oral slope factor (mg·kg^−1^·day^−1^)^−1^	Inhalation slope factor (mg·kg^−1^·day^−1^)^−1^	Inhalation unit risk Factor URF per (f/cm^3^)^−1^
IRIS			2.3E-1^a^
CALEPA	1.90e-4	2.20e + 2	1.9^b^
NDEP			2.3E-1^c^ 6.3^d^

^a^According to US EPA IRIS (integrated risk information system), the URF value refers to a combination of lung cancer and mesothelioma model for the population

^b^According to CALEPA (The California Environmental Protection Agency (OEHHA) Office of Environmental Health Hazard Assessment’s Chronic Reference Exposure Levels (RELS)), the URF value was determined using mesothelioma incidence in non-smoking females only for its derivation

NDEP (Nevada Division of Environmental Protection) approach distinguishes between amphibole and chrysotile risks (Black et al. 2011):

^c^Chrysotile URF

^d^Amphibole URF

**Table 2:** The estimated air concentrations resulting in lifetime cancer risks of 10^−4^, 10^−5^, and 10^-6^ (EPA 1986)

Risk Level	Concentration
E-4 (1 in 10,000)	4E-4 f/cm^3^
E-5 (1 in 100,000)	4E-5 f/cm^3^
E-6 (1 in 1,000,000)	4E-6 f/cm^3^

The inhalation unit risk is defined as the upper-bound excess lifetime cancer risk to result from continuous exposure to an agent at a concentration of 1 μg/m^3^ in air. It means for instance that if the unit risk = 2 × 10^-6^ per f/cm^3^ in air, two excess cancer incidents (upper bound estimate) are expected to develop per 1,000,000 people if exposed daily for a lifetime to one fiber of asbestos in 1 cm^3^ of air (EPA 1986).

According to the IRIS sources, the asbestos unit risk is based on fiber counts made by phase contrast microscopy PCM. Thus, the unit risk value derived in compliance with this approach should not be applied directly to measurements made by other analytical techniques.

The excess lifetime cancer risk *ELCR* has been determined using chronic exposure concentrations *EC* calculated from Eq. (2). This approach determines the probability of developing cancer over the lifespan at a given exposure level. In the case of using a procedure which involves calculation of lifetime average daily doses (LADD) and cancer slope factors (CSF), the obtained risk ELCR results are similar. ELCR is expressed by a value representing the number of extra cancer cases expected in a given number of people, on exposure to a carcinogen at a stated dose. ELCRs were calculated from the following:

1 $$ \mathrm{ELCR}=\mathrm{E}\mathrm{C}\times \mathrm{U}\mathrm{R}\mathrm{F} $$

where

EC: Chronic exposure concentration (averaged over a 70-year lifetime) [f/cm^3^]—for explanation see the Conclusions.
URF: Unit risk factor for asbestos inhalation [(f/cm^3^)^−1^].

For the risk assessment, US EPA IRIS unit risk factor of 0.23 per f/cm^3^ was applied, as the value consistent with the analyses of the air from the sites considered.

## Results and discussion

### Exposure assessment

**Fig. 1:**
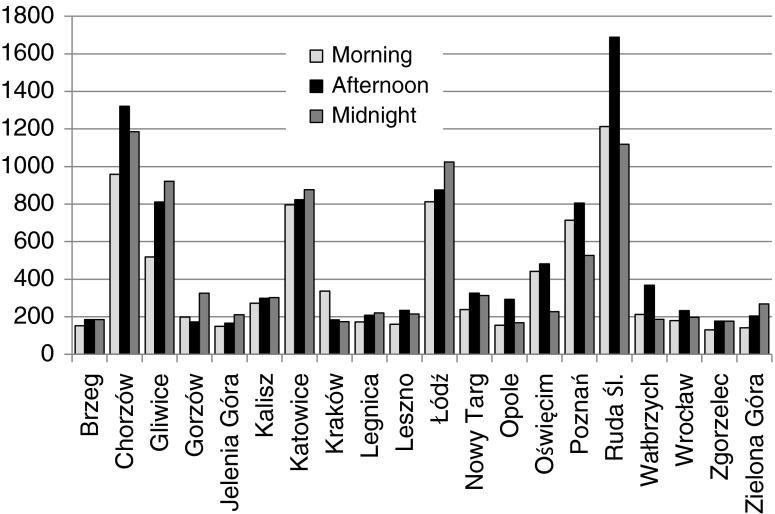
Concentration of asbestos fibers (Ca) in the ambient air in the south-west Poland towns at different times of the day [f/m^3^]

The exposure to asbestos airborne fibers depends on their concentration, the population age, and the applied exposure scenario. The fiber concentration in the air in each town was analyzed after the repeated triple sampling. The values obtained differed significantly, depending on the site and sampling time of day. The lowest air pollution was observed in small towns (i.e., 141–269 *f*/m^3^ in Zgorzelec), while in bigger cities, it was much higher. In Chorzów and Łódz for instance, it ranged between 800 and 1300 *f*/m^3^. Absolutely highest values were measured in Ruda Ślaska where the concentration reached almost 1700 *f*/m^3^. Generally, the increased levels of the pollution were noticed during the rush hours. On the other hand, the fiber concentration in the air was the lowest in the early morning. Figure 1 presents the maximum observed concentrations at particular sites.

Generally, from among 60 air samples taken, in 21 samples, that is in 35 %, the fiber concentration was below 200 *f*/m^3^. Only in three spots it exceeded 1000 *f*/m^3^.

The presented health risk assessment was carried out for the residential and occupational scenarios, that is for people who are exposed to the polluted air over 24 h a day and 8 h a day, respectively. Four age groups were considered in the scenario of residents involving children and adults (0– < 2 years, 2– < 6 years, 6– < 16 years and 16– < 30 years).

In terms of exposure duration (*ED*), the resident is assumed to live in the same home for 30 years, so residential exposure durations applied are 30 years, not a full lifetime of 70 years (EPA 1991). On the other hand, in the occupational scenario, it was assumed that exposure of adult employees starts with the age of 18 years and ends with the age of 55 years. The number of work days in a year was applied 220. The respective exposure duration for all the subpopulation groups and scenarios is given in Table 3.

**Table 3:** Population age division and exposure duration applied for risk assessment in residential and occupational scenarios

Scenario	Age range (years)	Exposure duration (years)
Residents	0– < 2	2
	2– < 6	4
	6– < 16	10
	16– < 30	14
Employees	18– < 55	37

It would be expected that entrainment of asbestos to the air depends, among other things, on traffic intensity, wind speed, and air humidity. It is obvious that the concentration is minimal on rainy days. This work assumes that the entrained fiber concentrations depend only on the traffic intensity not on the weather, as similar weather conditions were chosen for sampling. It was also assumed that residents are present at the spots for 24 h/day.

For the health risk assessment involving UCR values, the so called chronic concentrations have to be calculated. The following airborne asbestos inhalation exposure algorithm was used to determine chronic concentrations exposing all the age groups of receptors (Braun 2005; Lytle and Woo 2007)

2 $$ \mathrm{E}\mathrm{C}=\mathrm{C}\mathrm{a}\times \mathrm{E}\mathrm{T}\times \mathrm{E}\mathrm{F}\times \mathrm{E}\mathrm{D}/\mathrm{A}\mathrm{T} $$

where

EC: Chronic exposure concentration (averaged over a 70-year lifetime) [f/cm^3^].
Ca: Asbestos concentration in fibers per cubic centimeter [f/cm^3^].
ET: Exposure time in hours/day. It is 24 h a day in the case of the residents’ scenario and 8 h a day in the occupational scenario.
EF: Exposure frequency in days/year. In the resident scenario, it is 365 days a year. In the occupational scenario, it is (8 h/24 h) × 220 days a year (220 work days in the year was assumed and adults only are considered).
ED: Exposure duration in years. The value of ED depends on the age group. For the children from the group 0–2, it is 2 years, for the 2–6 group, it is 4 years. For adults 6–16 group, exposure duration = 10 years and for the 16–30 group, it is 14 years.
AT: Averaging time of 24 h/day × 365 days/year × 70 years (lifetime) = 613 200 h.

The EC values calculated for the considered scenarios, age populations, and sites are in Tables 4 and 5.

**Table 4:** The chronic exposure concentrations (EC) calculated for residents’ exposure, using maximum and minimum fiber counts [f/cm^3^]

Town	EC_0-2_		EC_2-6_		EC_6-16_		EC_16-30_	
	Min	Max	Min	Max	Min	Max	Min	Max
Brzeg	4.34E-06	5.29E-06	8.69E-06	1.06E-05	2.17E-05	2.64E-05	3.04E-05	3.70E-05
Chorzów	2.74E-05	3.77E-05	5.48E-05	7.54E-05	1.37E-04	1.89E-04	1.92E-04	2.64E-04
Gliwice	1.48E-05	2.63E-05	2.96E-05	5.27E-05	7.40E-05	1.32E-04	1.04E-04	1.84E-04
Gorzów	4.91E-06	9.31E-06	9.83E-06	1.86E-05	2.46E-05	4.66E-05	3.44E-05	6.52E-05
Jelenia G.	4.29E-06	6.03E-06	8.57E-06	1.21E-05	2.14E-05	3.01E-05	3.00E-05	4.22E-05
Kalisz	7.80E-06	8.63E-06	1.56E-05	1.73E-05	3.90E-05	4,31E-05	5.46E-05	6.04E-05
Katowice	2.27E-05	2.51E-05	4.55E-05	5.01E-05	1.14E-04	1,25E-04	1.59E-04	1.75E-04
Kraków	4.97E-06	9.60E-06	9.94E-06	1.92E-05	2.49E-05	4,80E-05	3.48E-05	6.72E-05
Legnica	4.94E-06	6.29E-06	9.89E-06	1.26E-05	2.47E-05	3,14E-05	3.46E-05	4.40E-05
Leszno	4.60E-06	6.69E-06	9.20E-06	1.34E-05	2.30E-05	3,34E-05	3.22E-05	4.68E-05
Łódź	2.32E-05	2.93E-05	4.64E-05	5.85E-05	1.16E-04	1,46E-04	1.62E-04	2.05E-04
Nowy Targ	6.80E-06	9.31E-06	1.36E-05	1.86E-05	3.40E-05	4,66E-05	4.76E-05	6.52E-05
Opole	4.43E-06	8.37E-06	8.86E-06	1.67E-05	2.21E-05	4,19E-05	3.10E-05	5.86E-05
Oświęcim	6.51E-06	1.37E-05	1.30E-05	2.75E-05	3.26E-05	6,87E-05	4.56E-05	9.62E-05
Poznań	1.51E-05	2.30E-05	3.01E-05	4.60E-05	7.53E-05	1,15E-04	1.05E-04	1.61E-04
Ruda Śl.	3.19E-05	4.82E-05	6.39E-05	9.65E-05	1.60E-04	2,41E-04	2.24E-04	3.38E-04
Wałbrzych	5.34E-06	1.05E-05	1.07E-05	2.10E-05	2.67E-05	5,26E-05	3.74E-05	7.36E-05
Wrocław	5.11E-06	6.66E-06	1.02E-05	1.33E-05	2.56E-05	3,33E-05	3.58E-05	4.66E-05
Zgorzelec	3.71E-06	5.06E-06	7.43E-06	1.01E-05	1.86E-05	2,53E-05	2.60E-05	3.54E-05
Zielona G.	4.03E-06	7.69E-06	8.06E-06	1.54E-05	2.01E-05	3,84E-05	2.82E-05	5.38E-05

**Table 5:** The chronic exposure concentrations (EC) calculated for occupational exposure, using maximum and minimum fiber counts [f/cm^3^]

Town	EC_18-55_		Town	EC_18-55_	
	Min	Max		Min	Max
Brzeg	1.61E-05	1.96E-05	Łódź	8.62E-05	1.09E-04
Chorzów	1.02E-04	1.40E-04	Nowy Targ	2.53E-05	3.46E-05
Gliwice	5.50E-05	9.79E-05	Opole	1.65E-05	3.11E-05
Gorzów	1.83E-05	3.46E-05	Oświęcim	2.42E-05	5.11E-05
Jelenia G.	1.59E-05	2.24E-05	Poznań	5.60E-05	8.55E-05
Kalisz	2.90E-05	3.21E-05	Ruda Śl.	1.19E-04	1.79E-04
Katowice	8.45E-05	9.31E-05	Wałbrzych	1.99E-05	3.91E-05
Kraków	1.85E-05	3.57E-05	Wrocław	1.90E-05	2.47E-05
Legnica	1.84E-05	2.34E-05	Zgorzelec	1.38E-05	1.88E-05
Leszno	1.71E-05	2.49E-05	Zielona G.	1.50E-05	2.86E-05

The above chronic exposure concentrations were then used for calculations of excess lifetime cancer risks for the four age groups of residents and employees exposed, using the formula (1).

### Risk characterization

**Fig. 2:**
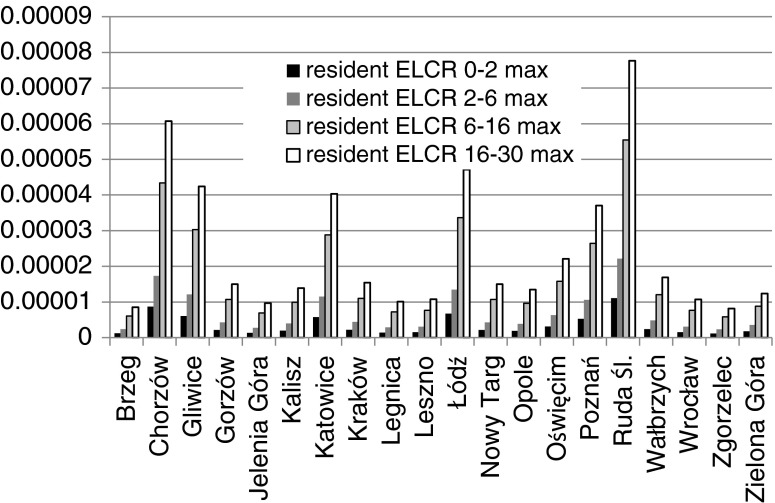
Excess lifetime cancer risks ELCR for the considered age groups and sites in the residents’ scenario

**Fig. 3:**
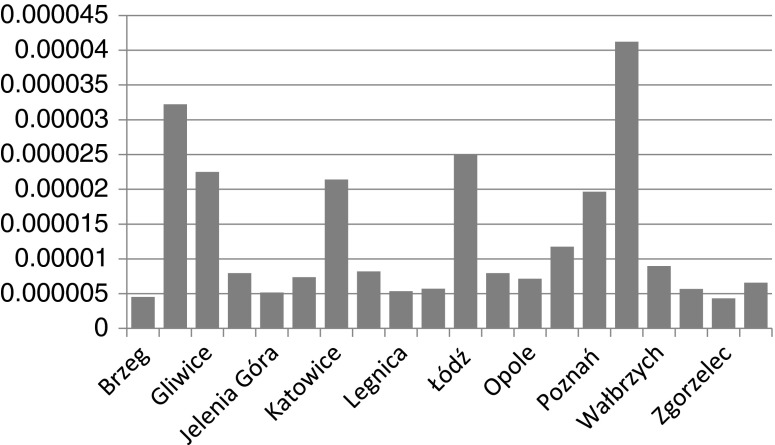
Excess lifetime cancer risks ELCR for the occupational scenario

The ELCRs estimated using the IRIS inhalation unit risk factor are presented in Figs. 2 and 3.

As can be seen from the Fig. 2, ELCR values for children from 0–2 and 2–6 years old groups are rather insignificant or moderate. For older age groups, that is for 6–16 and 16–30 years old populations, the maximum ELCR values exceed in many towns 1E-05. They may arouse a concern in case of Ruda Śl. and Chorzów, particularly when older children and adults are considered. In the original guidelines developed by Superfund, a carcinogenic risk range of 1 × 10^−4^ to 1 × 10^−7^ modified to 1 × 10^−4^ to 1 × 10^−6^ was recommended as a target risk level. Thus, the ambient chemical concentrations should be reduced to the levels providing at least the risk within the above mentioned limits (EPA 2015; Kelly 1991).

The National Contingency Plan in the USA designated 10^−6^ as a starting point for discussion of acceptable target risk at a site or as a “point of departure” (EPA 2015a). This problem has generated a lot of debate in scientific papers, and it still arouses controversies. Nevertheless, 10^−6^ is now generally regarded by literature as acceptable and safe (Callahan 2004).

The highest carcinogenic risk occurs in the resident scenario for adults from the 0–30-years age group. When site is concerned, the highest risks are observed in the towns of Ruda Sl., Chorzów, Łódz, Katowice, and Gliwice. Generally, the calculated risks are lowest in small towns with rather low traffic intensity.

The lifetime excess cancer risk for an adult in the resident scenario ELCR_0–30_ is composed of the sub-population risks. Thus,

3 $$ {\mathrm{ELCR}}_{0\hbox{--} 30}={\displaystyle \sum \mathrm{ELCRi}}={\mathrm{ELCR}}_{0\hbox{-} 2}+{\mathrm{ELCR}}_{2\hbox{-} 6}+{\mathrm{ELCR}}_{6\hbox{-} 16}+{\mathrm{ELCR}}_{16\hbox{-} 30} $$

The *ELCR*_0–30_ represents the lifetime probability of cancer incidence above the natural level in particular site, related to an adult resident of the age 30 years. The calculated maximum value of ELCR_0–30_ reaches 1.66E-04 in the case of Ruda Slaska, which exceeds the tolerable level. On the other hand, the real values are much lower because the asbestos fibers’ concentrations, the people are exposed to, are lower beyond the rush hours.

It should be stressed, that in all age populations, the risks exceed the acceptable level 1E-06. In the case of some areas of maximum values, the situation remains worrying and has to be constantly monitored. When the occupational scenario is considered, almost all values approach the levels around 1E-06 to 5E-06 which can be considered safe.

## Uncertainty of risk assessment

Evaluation procedure of the health risk to people exposed to asbestos in the environment assumes certain simplifications and ambiguities, due to which the obtained results cannot be regarded as definite and absolute. The procedure presented is based on the conservative approach to risk assessment, which tends rather to overestimate than underestimate the risk. The main factors contributing to assessment uncertainty are as follows:

- The exposure parameters used in these risk calculations (hours/day, days/year) were based on the assumption that the residents stay all the time at the area where they live. It is also obvious that a substantial period of time they remain in their homes where indoor concentrations are certainly different from the outside ones.
- It is obvious that the concentrations of the asbestos fibers in the air will not remain at a constant level over the whole lifetime of the individuals belonging to a given population. They fluctuate over a day and are highest during the rush hours. There are also uncertainties regarding the concentrations employed to determine the chronic exposure concentrations. Even though the samples were collected in similar weather conditions, the results must be interpreted with caution, because the sampling conditions were certainly not fully uniform at all sampling sites. The concentrations may vary depending on many unforeseen parameters, which can lead to some uncertainty in the derived ELCRs.
- The minimum and maximum fibers’ concentrations for all sites were used in the calculations, so the real risks should lie between the extreme values presented in the graphs.
- No distinction between different types of asbestos fibers (chrysotile, crocidolite) present in the air was made during the measurements which involves additional uncertainty associated with differences in the health effects caused by these kinds of asbestos. Also, PCM measurement method could be associated with some uncertainty as it detects all kind of airborne fibers in the environment.
- There is lack of data related to confounding factors such as smoking habits of the residents which could synergically increase the ELCRs calculated.

## Conclusions

Although the concentrations of the asbestos fibers in air do not exceed, except for three spots, the threshold value of 1000 fibers per one m^3^ generally agreed as an unofficial standard for the municipal air, the concentrations of the fibers in the centers of many towns concerned are quite high. Chronic exposure concentrations were calculated for adults and children for residents’ and occupational scenarios based on the minimum and maximum asbestos fiber concentrations in the examined sites.

It was proved that the calculated excess risks of cancer for all age groups exceed 10^−6^. Asbestos cancer risks for adult residents were all less than 1E-04 (100 in a million) even for maximum air concentrations, which could be still tolerable. For children 0–2 years old age group the risks slightly exceeded 1E-06.

The ELCR_0–30_ representing the lifetime mortality risk for lung cancer among adult residents is less than 1E-04, except for Ruda Sl. and Chorzów, where the maximum risks are 1.66E-04 and 1.30E-04, respectively. In fact, the real values should be lower because the maximum short time fibers’ concentrations in the air were applied to the calculations, not the real ones, which remain unknown.

The maximum occupational cancer risks calculated for all the sites are between 4.32E-06 and 4.12E-05 while the minimum ones between 3.18E-06 to 2.73E-5. According to the applied standards, these values are classified rather to moderate levels.

For all exposure scenarios assuming the maximum asbestos fibers’ concentration, the estimated excess lifetime cancer risks were within or they insignificantly exceeded the risk range recommended by EPA. The results obtained require further sampling to confirm or exclude the risk levels approaching 1E-04.

The conclusions of the cancer risk assessment must be interpreted with caution. It is important to point out that there is no evidence of a safe threshold for the carcinogenic effects of asbestos, and an increased cancer risk has been observed in populations exposed to very low concentrations of asbestos fibers. Taking that into consideration, it is recommended to reduce exposure as much as possible.

## References

[CR1] Agency for Toxic Substances and Disease Registry ATSDR (2001). Toxicological Profile for Asbestos, September 2001, http://www.atsdr.cdc.gov/ToxProfiles/tp61.pdf. Accessed 15 Aug 2014. 37983317

[CR2] Agency for Toxic Substances and Disease Registry ATSDR (2005). *Public Health Assessment Guidance* Manual, 2005 Update, http://www.atsdr.cdc.gov/hac/PHAManual/toc.html. Accessed 08 Oct 2014.

[CR3] American cancer Society ACS – Asbestos http://www.cancer.org/cancer/cancercauses/othercarcinogens/intheworkplace/asbestos. Accessed 7 Apr 2015.

[CR4] ATON (2014). Neutralization of asbestos waste, http://oferta.aton.com.pl/oferta/produkty/typoszereg-aton-ht/aton-hr-a, Accessed 1 Oct 2014.

[CR5] Black, P., Balshi, M., & Perona, R. (2011). Technical guidance for the calculation of asbestos related risk in soils for the Basic Management Incorporated (BMI) complex and common areas, *NDEP Guidance for Asbestos-Related Risk.*

[CR6] Block, J. (1998). Composition and method to remove asbestos, Pat. USA No. 5753031, 19.05.1998.

[CR7] Braun, R. (2005). Asbestos Exposure and Human Health Risk Assessment, Asbestos Air Sampling, Nov. 7, 2005, http://www.epa.gov/Region9/toxic/noa/clearcreek/pdf/CCMA_HHRATechMemo11-2004Sampling_approval.pdf. Accessed 10 Aug 2014.

[CR8] Breslow, L. et al. (1984). National Academy Press, Committee on Nonoccupational Health Risks of Asbestiform Fibers, Board on Toxicology and Environmental Health Hazards,Commission on Life Sciences,Division on Earth and Life Studies,National Research Council, Washington DC, 1984, Asbestiform Fibers: Nonoccupational Health Risks; https://books.google.pl/books?id=8wiBqc6iz0cC&pg=PA9&lpg=PA9&dq=uncertainty+in+conversion+from+fiber+measurement+to+mass+measurement&source=bl&ots=FSggJUB3Am&sig=KpAnmJvJxqKZGtn1mxugc6OQSU4&hl=pl&sa = X&ei=DUgmVeeRJImNsAGRqILoCw&ved=0CCUQ6AEwAA#v=onepage&q=uncertainty%20in%20conversion%20from%20fiber%20measurement%20to%20mass%20measurement&f=false

[CR9] Brownson, R. D., Warner, K. K., & Rosenthal, J. E. (2012). Current and Historical American Asbestos Regulations, Brownson & Ballou, PLLP, *Monaldi Archives for Chest Disease* 1998 *53*(No. 2):181–85, 2012 update 9689805

[CR10] Callahan B (2004). Review of the army's technical guides on assessing and managing chemical hazards to deployed personnel.

[CR11] Debailleul, G. (2002). Process for the treatment of waste containing asbestos. Pat. USA No. 6391271, 21.05.2002.

[CR12] Environmental Protection Agency (2015b). Integrated risk information system. http://www.epa.gov/iris/subst/0371.htm. Accessed 31 Mar 2015.

[CR13] Environmental Protection Agency (2015a). Regional removal management levels for chemicals. January 2015, http://www.epa.gov/region4/superfund/programs/riskassess/rml/rmlfaq.html

[CR14] Environmental Protection Agency EPA (1980). Superfund – Comprehensive Environmental Response, Compensation and Liability Act. http://www.epa.gov/superfund. Accessed 1 Oct 2014.

[CR15] Environmental Protection Agency EPA (1986). Risk assessment for carcinogens. http://www.epa.gov/ttnatw01/toxsource/carcinogens.html. Accessed 1 Oct 2014.

[CR16] Environmental Protection Agency EPA (1989). Risk assessment guidance for superfund, Vol. I, Human Health Evaluation Manual, Part A. http://www.epa.gov/oswer/riskassessment/ragsa/pdf/rags-vol1-pta_complete.pdf. Accessed 1 Oct 2014.

[CR17] Environmental Protection Agency EPA (1991). Risk Assessment guidance for superfund, Vol. I, Human Health Evaluation Manual, Supplemental Guidance “Standard Default Exposure Factors”, OSWER Directive: 9285.6-03, March 25, 1991. http://rais.ornl.gov/documents/OSWERdirective9285.6-03.pdf. Accessed 1 Oct 2014.

[CR19] Environmental Protection Agency EPA (2011a). 2011 Edition of the drinking water standards and health advisories. EPA 820-R-11-002. Washington DC http://water.epa.gov/action/advisories/drinking/upload/dwstandards2011.pdf. Accessed 11 Oct 2014.

[CR18] Environmental Protection Agency EPA (2011b). Exposure assessment. http://cfpub.epa.gov/ncea/iris/index.cfm?fuseaction=iris.showSubstanceList. Cited 10 Aug 2011 Accessed 1 Oct 2014.

[CR20] Environmental Protection Agency, Risk Communication, Attachment - 6: Useful Terms and Definitions for Explaining Risk http://www.google.pl/url?sa=t&rct=j&q=&esrc=s&source=web&cd=2&cad=rja&uact=8&ved=0CC0QFjAB&url=http%3A%2F%2Fwww.epa.gov%2Fsuperfund%2Fcommunity%2Fpdfs%2Ftoolkit%2Frisk_communication.pdf&ei=Be4wVbOUM5WwaZjigNgP&usg=AFQjCNGGPHMYxF_dqRMjrbCsz5kEVKo8sg&sig2=_2_YwC61ODtZENtrV-FPBA). Accessed 11 Apr 2015.

[CR21] Integrated Risk Information System IRIS (2011). http://www.epa.gov/iris/subst/0371.htm. Accessed 11 Oct 2014.

[CR22] International Agency for Research on Cancer IARC (2011). Agents Classified by the IARC Monographs, Vol. 1–102. http://monographs.iarc.fr/ENG/Classification/ClassificationsAlphaOrder.pdf. Cited 17 Aug 2011 Accessed 11 Oct 2014.

[CR23] Kelly, K. E. (1991). The Myth of 10-6 as a Definition of Acceptable Risk, 84th Annual Meeting, Air & Waste Management Association, Vancouver, B.C., Canada, 16-21 June, 1991 http://www.google.pl/url?sa=t&rct=j&q=&esrc=s&source=web&cd=4&cad=rja&uact=8&ved=0CD0QFjAD&url=http%3A%2F%2Fnews.heartland.org%2Fsites%2Fall%2Fmodules%2Fcustom%2Fheartland_migration%2Ffiles%2Fpdfs%2F17603.pdf&ei=kAkpVZafD9PcaJrHgsgD&usg=AFQjCNGIODXrfACJX6QGDKi_1i6max7MvA&sig2=Kb6Fy_lBAQvQwHqNPBOTpA.

[CR24] Krakowiak E, Górny R, Cembrzyńska J, Sąkol G, Boissier-Draghi M, Anczyk E (2009). Environmental exposure to airborne asbestos fibres in a highly urbanized city. Annals of Agricultural and Environmental Medicine.

[CR25] London Hazards Centre LHC (1995). Interchange studios, the asbestos hazards handbook. http://www.lhc.org.uk/members/pubs/books/asbestos/asb_toc.htm. Accessed 12 Sept 2014

[CR26] Lytle, G., & Woo, C. (2007). Asbestos exposure and human health risk assessment, asbestos air sampling. EPA Region 9; Jere Johnson, WAM, Atlas Asbestos Mine Superfund Site, CCMA Human Health Risk Assessment. September 5.

[CR27] Ministry of Environment ME (1998). Poland, decree on maximal allowable concentrations of air polluting substances. *Journal of Laws of the Republic of Poland*, Dz.U. No. 55, item 355. 28 April 1998.

[CR28] Ministry of Labor and Social Policy MLSP (2002). Poland, Decree related to Maximal Allowable Concentrations and Intensity of Harmful Factors in Occupational Environment. *Journal of Laws of the Republic of Poland*, Dz.U. No. 217, item 1833.

[CR29] Mirick, W., & Forrister, W. (1993). Products for treating asbestos, Pat. USA No. 5258131, 2.11.1993.

[CR30] National Cancer Institute NCI (2011). Asbestos exposure and cancer risk. http://www.cancer.gov/cancertopics/factsheet/Risk/asbestos.

[CR31] Office of Environmental Health Hazard Assessment OEHHA (2011). Hot spots unit risk and cancer potency values. http://oehha.ca.gov/air/hot_spots/pdf/CPFs042909.pdf. Accessed 3 Jul 2014.

[CR32] O'Reilly K, Mclaughlin A, Beckett W, Sime P (2007). Asbestos-related lung disease. American Family Physician.

[CR33] Polish Standard PS (1984). Protection of air purity. Air sampling. General guidelines for atmospheric air sampling. PN-84/Z-04008.02.

[CR34] Polish Standard PS (1988). Protection of air purity. Investigation of asbestos contents. Determination of number of respirable asbestos fibers on work stands by optical microscopy method. PN-88/Z-04202/02.

[CR35] Pritchett, J. (1997). Method for abating bio-hazardous materials found in coatings, Pat. USA No. 569812, 9.12.1997.

[CR36] Programme for Asbestos Abatement in Poland 2009-2032 (2010). Annex to the Resolution No. 39/2010 of the Council of Ministers of 15 March 2010, Warsaw.

[CR37] Risk Assessment Information System RAIS (2011). Basic information for asbestos (in fibers). http://rais.ornl.gov/tools/profile.php?analysis=Asbestos (in fibers). Accessed 21 Sept 2014.

[CR38] Salvatore, C., Scheff, P., & Sokas, R. (2006). *Determination of Asbestos Contamination in Beach Nourishment Sand, Final Report of Findings*, University of Illinois at Chicago.

[CR39] Trefler, B., Pawełczyk, A., Zwozdziak, J., & Czarny, A. (2003). Method of waste free utilization of asbestos and asbestos containing materials. Polish Pat. Appl No. P-359958, 5.05.2003.

[CR40] Trefler B, Pawełczyk A, Nowak M (2004). The waste free method of utilizing asbestos and the products containing asbestos. Polish Journal of Chemical Technology.

[CR41] Wagner JC, Sleggs CA, Marchand P (1960). Diffuse pleural mesothelioma and asbestos exposure in the North Western Cape Province. British Journal of Industrial Medicine.

[CR42] World Health Organization WHO (2014). Asbestos: elimination of asbestos-related diseases http://www.who.int/mediacentre/factsheets/fs343/en/. Accessed 21 Sep 2014.

[CR43] Zaremba T, Krząkała A, Piotrowski J, Garczorz D (2010). Study on the thermal decomposition of chrysotile asbestos. Journal of Thermal Analysis and Calorimetry.

[CR44] Zaremba T, Krząkała A, Piotrowski J, Garczorz D (2011). Investigations of chrysotile asbestos application for sintered ceramics obtaining. Materialy Ceramiczne (Ceramic Materials).

